# Association between dental caries experience and salivary profile among autoimmune thyroid disease subjects - a cross-sectional comparative study

**DOI:** 10.12688/f1000research.135684.1

**Published:** 2023-07-14

**Authors:** Aparna K S, Manjunath P Puranik, Uma S R

**Affiliations:** 1 Department of Public Health Dentistry, Manipal College of Dental Sciences, Mangalore, Manipal Academy of Higher Education, Manipal, Karnataka, 575001, India; 2 Public Health Dentistry, Government Dental College and Research Institute Bangalore, Bangalore, Karnataka, 560002, India

**Keywords:** autoimmune thyroid disease, buffering capacity, dental caries, flow rate, pH, saliva, salivary viscosity

## Abstract

**Background:** Autoimmune thyroid disease (AITD) is an inflammatory condition that primarily affects women between the ages of 30 and 50. It has been hypothesised that AITD causes salivary glands to produce less saliva due to its endocrine effects. Studies showing the effect of AITD on salivary glands are very scarce. The aim of this study was to compare AITD patients with controls who were of a similar age and gender in terms of salivary metrics and the prevalence of dental caries.

**Methods:** 200 subjects in Bangalore city (100 AITD subjects and 100 healthy subjects as controls) participated in a cross-sectional study. Subjective oral dryness was assessed using Farsi’s criteria. Salivary parameters and caries were assessed using GC Saliva-Check BUFFER kit and WHO caries criteria respectively. Descriptive and analytical statistics were done. Significant data was defined as a *p* value of <0.05.

**Results**: When compared to controls, the AITD group had substantially more subjective mouth dryness and dental caries. Unstimulated (USFR) and stimulated salivary flow rate (SFR), pH and buffering capacity were reduced in AITD group whereas viscosity was high. There was a significant negative correlation between USFR, SFR, subjective oral dryness and dental caries. In a linear regression, there was no association between age, gender, socioeconomic status (SES), thyroid stimulating hormone (TSH), salivary viscosity, pH, buffering capacity and dental caries. Dental caries, USFR and SFR demonstrated a substantial relationship.

**Conclusions**: The present research may indicate an underlying association between thyroid and salivary gland dysfunction resulting in oral dryness and high dental caries experience.

## Introduction

The immune system is dysregulated in autoimmune thyroid disease (AITD), which leads to an attack on the thyroid gland. Its two primary clinical symptoms are Graves disease (GD) and Hashimoto’s thyroiditis (HT). An estimated 5% of the general population is thought to be affected by HT and GD. AITD develops when genetically susceptible people lose their tolerance to thyroid antigens in conjunction with environmental factors such altered microbiota. Both symptoms are marked by aberrant thyroid functioning and T and B cell infiltration into the thyroid that is responsive to thyroid antigens. ^1^

AITD is unique to thyroid gland and women are affected 4–10 times more frequently than males. ^2^ The most prevalent forms, Hashimoto’s thyroiditis (HT) and Graves’ disease (GD), have many immune characteristics in common. The primary characteristic of AITD is the emergence of antibodies against thyroid peroxidase (TPO), thyroglobulin (TG), and thyroid stimulating hormone receptor (TSH-R). ^3^

A combination of main and minor salivary gland secretions, gingival crevicular fluid, cellular debris, and bacteria are all present in saliva. Saliva serves a wide range of purposes, including the defence of the mouth cavity, digesting owing to salivary amylase, immunity to microbes, and, most critically, for use in diagnostic activities. The use of saliva in diagnostic procedures is expanding since it is simple, non-invasive, and affordable. Enzymes, hormones, antibodies, antibacterial components, growth factors, and other substances that enter from the blood through the gaps between cells can all be found in saliva. Some of the conditions for which saliva has significant diagnostic potential are oncological, endocrine, cardiovascular, rheumatic, autoimmune, neurological, or viral illnesses. ^1^

Dental caries is seen as a complex microbiological illness. It is a multifactorial disease of the teeth that results in a localised loss of tooth structure. It is caused by the interaction of dietary carbohydrates, tooth substrate, and cariogenic bacteria in the dental biofilm, which produces acid after carbohydrate fermentation and causes changes in the pH of the biofilm, which leads to mineral loss (demineralization) as a result of disturbances in the physiologic equilibrium between the biofilm and tooth. Previous studies have discovered an increase in the prevalence of dental caries in people with thyroid dysfunction, either as a result of the disease process itself, as a result of the surgical treatment (thyroidectomy), or as a result of medication taken that worsens oral and dental conditions. ^4^

The amount and quality of saliva generated are both impacted by AITD, which either directly or tangentially affects salivary gland secretory function. The primary determinants of salivary defence are its composition, buffering ability, pH and flow rate. Saliva production, when reduced, has an impact on mouth health and can cause complications like oral candidiasis, gum disease, tooth cavities, and other pharyngeal and oral disorders. ^3^

Previous studies have examined the major involvement of the salivary glands in instances with AITD, which suggests that the development of thyroid and salivary gland immunological diseases may have a same mechanism. ^4^ According to animal research, thyroid malfunction alters basal metabolic rate, which may therefore have an impact on the salivary gland’s secretary unit. ^5^

There are very few studies demonstrating how AITD affects the salivary ducts. Because of this, the following research topic guided the execution of this study: Do AITD participants’ experiences with tooth caries and their salivary characteristics correlate? The link between dental caries history and salivary profile among AITD patients was postulated to exist. The objectives were to evaluate the relationship between tooth caries and salivary profile in AITD individuals and to contrast it with controls who were of a similar age and gender.

## Methods

### Study design and population

In Bangalore metropolis, a cross-sectional research study involving AITD participants was carried out from June to August (pilot) and August to September (full study) in 2018.

### Ethics

The Institutional Ethical Committee (GDCRI/IEC-ACM(2)/9/2018-19, dated 21/08/2018) granted their approval for this study. The appropriate approval was received from the hospital’s administrators. The pilot study was conducted prior to ethical approval as this is part of the approval application process. After explicitly outlining the study’s goal and methodology, participants gave their written informed consent. Prior to the commencement of the research, the investigator was trained and calibrated to guarantee reliability (k = 0.80).

### Questionnaire

The Farsi oral dryness test, which consists of four queries and answer options, was used. ^6^ During the pilot research, readability and understanding were evaluated. Necessary corrections and modifications were made. Internal consistency (α) was found to be good (0.87).

### Sample size

To determine the sample number and assess the study’s viability, a pilot study with 10 participants was carried out. The sample size was determined based on the frequency of dental caries using the following formula:

$$N=\frac{Z_{\alpha /2^{2}}\times P\left(1-P\right)\times D}{E^{2}}$$

“Where P = Prevalence of reduced unstimulated salivary flow rate i.e., 80.0%, statistical power = 80%, Z _α_ = 1.96 at 95% confidence interval, E = margin of error-10%, Design effect, D = 1”

$$N=\frac{{\left(1.96\right)}^{2}\times 0.80\left(1-0.80\right)\times 1}{{\left(0.080\right)}^{2}}=96.04$$

The sample size obtained was 96.04 which was rounded up to 100. Hence, 100 AITD subjects and 100 healthy, age- and gender-matched subjects (subjects’ companions) were chosen based on the eligibility criteria.

### Eligibility criteria

*AITD subjects*

Inclusion criteria

The inclusion criteria were subjects aged 18 years and above diagnosed with autoimmune thyroid disease (both hypo and hyper AITD), and subjects with no other systemic illness.

Exclusion criteria

The exclusion criteria were subjects who smoked or drank alcohol, subjects with a history of/undergoing radio-active iodine, and subjects taking any xerogenic medication.

*Healthy subjects*

Inclusion criteria

The inclusion criteria were those 18 years or older without systemic illness, whose age and gender matched subjects in the AITD group.

Exclusion criteria

Excluded from the study were subjects with any habits like smoking and alcohol consumption.

### Data collection

Based on inclusion and exclusion factors, research participants were chosen at random from a hospital that was randomly selected from the list of hospitals in Bangalore. ^7^ The study period was from June to September 2018. Patients who met the eligibility criteria were approached in the hospital based on the medical records and their bystanders were included as the control group. The participants were given an information sheet outlining the purpose of the study, the specifics of the research study, and the processes, after which they consented to participate in the study. Their queries regarding the study were addressed. Written informed consent was acquired. The study’s participants were assured their right to withdraw and were free to leave at any moment. From the data that were accessible from the medical records, thyroid disorders were screened for in all research participants. Among 100 AITD subjects, equal distribution of hyper and hypo AITD were maintained. Most of them were diagnosed within the past year. Data regarding thyroid hormone levels were obtained from the hospital records. Increased blood levels of thyroid stimulating hormone (TSH) > 5mIU/l, low serum free tetraiodothyroxine (FT4) < 0.61 ng/dl, and elevated thyroid peroxidase (TPO) > 34 IU/ml were used to identify autoimmune hypothyroiditis. Based on reduced (TSH) < 0.3 mIU/l, TSH receptor antibody, and elevated FT4 > 2 ng/dl, autoimmune hyperthyroidism was identified. ^8^ Data were gathered by a single calibrated observer using an organised questionnaire, clinical evaluation, and recording by a trained assistant. In addition to demographic information, dentist appointments and oral care routines were logged. Socioeconomic status (SES) was evaluated using an adapted Kuppuswamy categorization system. ^9^

Throughout the research, infection control practises were followed. All the data were collected at one instance from each patient which comprised of demographic details, subjective assessment of saliva using Farsi’s questionnaire, dental caries examination and objective assessment of saliva using GC Saliva-Check BUFFER kit.

Demographic details were first collected by the principal examiner followed by the administration of Farsi’s questionnaire. Oral dryness was assessed using Farsi’s oral dryness questionnaire which comprised of 4 items where a response of ‘Yes’ was scored as 1 and ‘No’ was scored as 0. Positive response (Yes) was indicative of subjective dryness. Utilizing WHO 2013 caries criteria, ^10^ clinical evaluation of oral caries was conducted.

The criteria for diagnosing a tooth status and the coding are as follows:

“0 = Sound

1 = Caries

2 = Filled with caries

3 = Filled, no caries

4 = Missing due to caries

5 = Missing for any other reason

6 = Fissure sealant

7 = Fixed dental prosthesis/crown abutment, veneer, implant

8 = Unerupted

9 = Not recorded”

Clinical examination of dental caries was followed by saliva collection of each patient.

To avoid confounding brought on by diurnal fluctuation, saliva was taken from each subject at the same time of day (between 9 and 11 AM). Viscosity was assessed visually in the oral region by observing the consistency of unstimulated saliva. Normal viscosity was indicated by watery, transparent spit. Saliva that was sticky, foamy, and effervescent was a sign of increased viscosity.

Salivary parameters were assessed using GC Saliva-Check BUFFER kit (GC India Dental Pvt. Ltd.), comprised of graduated collecting cylindrical tube with pipettes, pH strips, buffer strips, colour indicator chart and paraffin wax. ^11^ Saliva was stimulated with paraffin wax. The participants in the research were instructed to chew on a portion of paraffin wax for 5 minutes while spitting into a gathering cup every 30 seconds. Patients were handed graded collecting cylindrical tubes and instructed to sit up straight for five minutes. They were told to spit into the conduit for five minutes without ingesting (drooling method). The salivary composition, which included USFR, SFR, pH, and buffering capacity, was estimated from the collected saliva. Normal range of USFR is 0.2-0.3 ml/ min and below 0.2ml/ min was considered as reduced salivary flow. Normal range of SFR is 1-3 ml/min and below 1ml/min was considered as reduced salivary flow.

The pH was measured using pH strips with unstimulated saliva. The participants in the research were told to expectorate any accumulated saliva into the gathering cup. For 10 seconds, the pH strip was inserted into the saliva. The strip’s hue was contrasted with the colour indicator chart, where green represented healthy saliva. Yellow and red colours indicated moderately and highly acidic saliva respectively. Buffering capacity was measured with stimulated saliva using buffer strips.

Stimulated saliva was drawn from the collection cup using a pipette and one drop was dispensed to each of the three test pads. The colour of the strip was compared with the colour indicator chart where green colour denoted normal/high buffering capacity. Yellow and red colours indicated low and very low buffering capacity respectively.

### Statistical analysis

The gathered information was put into a Microsoft Excel spreadsheet. The Statistical Package for Social Sciences was used to analyse the data (IBM SPSS Statistics V22.0). Statistics were analysed using both descriptive and inferential methods. Subgroup analysis was performed and analysis was done between autoimmune hyperthyroidism and autoimmune hypothyroidism. Pearson’s association, the unpaired t-test, the chi square test and Fisher exact test, as well as linear regression, were used. Dental caries was the dependent variable in a linear regression analysis with unstimulated saliva, stimulated saliva, salivary viscosity, salivary pH, and salivary buffering capacity as the independent factors. By using an online calculator, ^9^ Kuppuswamy’s salary categories were revised with the Consumer Price Index for Industrial Workers (CPI-IW) at 291 for June 2018. A 5% level of significance ( *p* < 0.05) was deemed acceptable.

## Results

This study included 100 AITD subjects and 100 healthy subjects (controls). The mean age for AITD group and control group were 32.20 ± 4.21years and 33.93 ± 5.55 years respectively. There was a higher proportion of female participants than males. The majority of the study subjects belonged to lower middle class (85.8%). Between the research groups, there was no statistically significant variation in terms of age ( *p* = 0.06), gender ( *p* = 0.30), or socioeconomic position ( *p* = 0.29) ( Table 1).

**Table 1.** Demographic characteristics of the study participants.

Demographic data		AITD group N = 100	Control group N = 100	*p* value
Age (years)	Mean ± SD	32.20 ± 4.21	33.93 ± 5.55	0.06
Gender	Males	35 (35.0)	42 (42.0)	0.30
	Females	65 (65.0)	58 (58.0)	
Socioeconomic status	Upper (I)	0 (0)	0 (0)	0.29
	Upper middle (II)	06 (6.0)	06 (6.0)	
	Lower middle (III)	79 (79.0)	86 (86.0)	
	Upper lower (IV)	15 (15.0)	08 (8.0)	
	Lower (V)	0 (0)	0 (0)	

The majority of research subjects (AITD: 75%, control: 68%) had never been to the dentist. The majority of those who had attended a dentist had done so within the previous year (AITD: 72%, control: 53.1%). Tooth extraction (AITD: 40%, control: 46.9%) was the most frequently reported dental procedure, and pain was the primary factor driving dental visits (AITD: 60%, control: 62.5%). Between the research groups, there was no statistically significant variation in the duration since last dental visit ( *p* = 0.05), the causes for the dental appointment ( *p* = 0.82), or the type of treatment received ( *p* = 0.76).

The majority of the AITD subjects (81%) were under anti-thyroid drugs for the past 1 year. Mean TSH values among AITD hyper and hypo groups were 0.20 ± 0.07 and 6.43 ± 1.30 respectively ( Table 2). Comparing the AITD group to the control group, a substantially greater percentage of respondents reported subjective dryness ( Table 3). Regarding subjective dryness, there was no statistically significant difference between the AITD hyper and hypo groups ( Table 4).

**Table 2.** History of autoimmune thyroid disease among study participants.

History		AITD group N = 100
AITD type	AITD hyperthyroid	50 (50.0)
	AITD hypothyroid	50 (50.0)
Duration of diagnosis	<6 months	20 (20.0)
	6 months-1 year	74 (74.0)
	1-2 years	04(4.0)
	>2 years	02(2.0)
Duration of antithyroid drugs usage (N = 81)	<6 months	20(24.7)
	6 months-1 year	54(66.7)
	1-2 years	04(4.9)
	>2 years	03(3.7)
Mean TSH (microgram/decilitre)	AITD hyperthyroid	0.20 ± 0.07
	AITD hypothyroid	6.43 ± 1.30

**Table 3.** Subjective oral dryness among study participants according to Farsi’s criteria.

Questions	Positive response to oral dryness		*p* value
	AITD group N = 100	Control group N = 100	
1. Does your mouth feel dry?	40 (40.0)	07 (7.0)	**<0.001**
2. Do you sip liquids to aid in swallowing dry food?	32 (32.0)	05 (5.0)	**<0.001**
3. Does your mouth feel dry when eating a meal?	30 (30.0)	03 (3.0)	**<0.001**
4. Does the amount of saliva in your mouth seem to be too little?	27(27.0)	03 (3.0)	**<0.001**

Bold values are significant ( *p* value < 0.05).

**Table 4.** Subjective oral dryness among AITD groups according to Farsi’s criteria.

Questions	Positive response to oral dryness		*p* value
	AITD Hyper n = 50	AITD Hypo n = 50	
1. Does your mouth feel dry?	17 (34.0)	23 (46.0)	0.09
2. Do you sip liquids to aid in swallowing dry food?	14 (28.0)	18 (36.0)	0.07
3. Does your mouth feel dry when eating a meal?	11 (22.0)	19 (38.0)	0.09
4. Does the amount of saliva in your mouth seem to be too little?	11 (22.0)	16 (32.0)	0.06

When compared to the control group, the amount of stimulated and unstimulated saliva was considerably lower in the AITD group. In comparison to the control group, a considerably greater percentage of research participants in the AITD group had increased salivary viscosity. When compared to the control group, salivary pH and buffering capacity were considerably lower in the AITD group ( Table 5). Regarding salivary measures, there was no statistically significant variation between the AITD hyper and hypo groups ( Table 6).

**Table 5.** Salivary parameters among study participants.

Salivary parameters	AITD group N = 100	Control group N = 100	*p* value
Unstimulated saliva (reduced)	62 (62.0)	09 (9.0)	**<0.001**
Stimulated saliva (reduced)	58 (58.0)	09 (9.0)	**<0.001**
Salivary viscosity (increased)	45 (45.0)	02 (2.0)	**<0.001**
Salivary pH (low)	47 (47.0)	04 (4.0)	**<0.001**
Salivary buffering capacity (low)	45 (45.0)	02 (2.0)	**<0.001**

Bold values are significant ( *p* value < 0.05).

**Table 6.** Salivary parameters among AITD groups.

Salivary parameters	AITD hyper n = 50	AITD hypo n = 50	*p* value
Unstimulated saliva (Reduced)	26 (52.0)	36 (72.0)	0.21
Stimulated saliva (Reduced)	25 (50.0)	33 (66.0)	0.18
Salivary viscosity (Increased)	20 (40.0)	25 (50.0)	0.31
Salivary pH (Low)	21 (42.0)	26 (52.0)	0.32
Salivary buffering capacity (Low)	20 (40.0)	25 (50.0)	0.59

Anti-thyroid medications did not significantly affect either unstimulated ( *p* = 0.82) or stimulated salivary flow ( *p* = 0.88), viscosity ( *p* = 0.19), pH ( *p* = 0.32), or buffering capacity ( *p* = 0.36), respectively).

When compared to the control group (56%), the AITD group (72%) experienced considerably more dental cavities. Mean number of DMFT, decayed teeth (DT) and missing (MT) teeth were significantly higher in AITD group (DMFT: 3.82 ± 1.36, DT: 3.18 ± 1.06, MT: 0.45 ± 0.83) than the control group (DMFT: 1.65 ± 0.86, DT: 1.25 ± 0.53, MT: 0.19 ± 0.59) ( *p* < 0.001). There was no significant difference between the mean numbers of filled (FT) teeth among the AITD group (0.19 ± 0.63) and the control group (0.21 ± 0.47) ( *p* = 0.15) ( Table 7). There was no statistically significant difference among the AITD hyper and hypo groups with regard to dental caries experience ( Table 8).

**Table 7.** Mean caries experience (DMFT) among study participants.

Caries experience	AITD group (Mean ± SD)	Control group (Mean ± SD)	*p* value
DT	3.18 ± 1.06	1.25 ± 0.53	<0.001
MT	0.45 ± 0.83	0.19 ± 0.59	**<0.001**
FT	0.19 ± 0.63	0.21 ± 0.47	0.15
DMFT	3.82 ± 1.36	1.65 ± 0.86	**<0.001**

Bold values are significant ( *p* value < 0.05).

**Table 8.** Mean caries experience (DMFT) among AITD groups.

Caries experience	AITD hyper (Mean ± SD)	AITD hypo (Mean ± SD)	*p* value
DT	2.56 ± 1.21	3.80 ± 1.43	0.12
MT	0.41 ± 0.78	0.51 ± 0.39	0.55
FT	0.16 ± 0.42	0.22 ± 0.79	0.63
DMFT	3.12 ± 1.75	4.52 ± 1.86	0.10

A significant negative correlation was observed between subjective mouth dryness and “USFR” (r = -0.586, *p* < 0.001) and DMFT and “USFR” (r = -0.368, *p* < 0.001). “SFR” had a significant negative correlation with subjective mouth dryness (r = -.524, *p* < 0.001) and DMFT (r = -0.373, *p* < 0.001) respectively ( Table 9).

**Table 9.** Correlation between USFR, SFR, subjective oral dryness and Dental Caries.

Variable	Correlation value (r)		*p* value
	USFR	SFR	
Subjective oral dryness	-.586 *	-.524 *	**<0.001**
DMFT	-.368 *	-.373 *	**<0.001**

* Significant ( *p* value < 0.05).

Bold values are significant ( *p* value < 0.05).

In the linear regression model, age (β **=** 0.115, R ^2^ = 0.016, *p* = 0.45), gender (β **=** 0.089, R ^2^ = 0.019, *p* = 0.44), socioeconomic status (β **=** 0.021, R ^2^ = 0.001, *p* = 0.86), thyroid stimulating hormone (β **=** -0.091, R ^2^ = 0.006, *p* = 0.43), salivary viscosity (β **=** 0.125, R ^2^ = 0.02, *p* = 0.74), pH (β **=** -0.919, R ^2^ = 0.01, *p* = 0.12) and buffering capacity (β **=** 0.778, R ^2^ = 0.03, *p* = 0.26) were not associated with dental caries. Unstimulated saliva (β **=** -0.523, R ^2^ = 0.43, *p* = 0.006) and stimulated saliva (β **=** -0.476, R ^2^ = 0.32, *p* = 0.003) showed a significant association with dental caries ( Table 10).

**Table 10.** Linear regression analysis with dental caries as dependent variable.

Independent variables	Standardized regression coefficients (β)	R ^2^	*p* value
Age	.115	0.016	0.45
Gender	.089	0.019	0.44
SES	.021	0.001	0.86
TSH	-.091	0.006	0.43
Unstimulated saliva	-.523	0.43	**0.006**
Stimulated saliva	-.476	0.32	**0.003**
Salivary viscosity	.125	0.02	0.74
Salivary pH	-.919	0.01	0.12
Salivary buffering capacity	.778	0.03	0.26

Bold values are significant ( *p* value < 0.05).

## Discussion

Autoimmune thyroiditis is a chronic disease in which the body interprets the thyroid glands and its hormones T3, T4 and TSH as threats. ^12^ Sex hormones play a big role in triggering or protecting from autoimmunity. Estrogen has the ability to enhance the inflammatory process of the immune system *and* could contribute to AITD. ^12^^,^^13^ The mean age of the AITD group was 32.20 ± 4.21 years which is similar to a previous study (30.06 years). ^5^ The majority of participants were female which is in line with two previous studies. ^5^^,^^14^

Due to the chronic nature of autoimmune illness, there will be significant financial expenses as well as a negative effect on the patient’s health and quality of life. Dental caries are more likely to occur when socioeconomic factors, lower levels of schooling, and lower revenue are combined. ^15^ Most of the research participants were from the lower middle class. Age, gender, and socioeconomic position among the research groups did not vary numerically significantly, indicating homogeneity.

Dental visits are of utmost importance to high-risk groups like autoimmune thyroid disease subjects as they are more prone to a variety of dental problems. The majority of the research participants had never been to a dentist. Most of the trips were motivated by symptoms (pain), which shows that the respondents did not fully understand the value of a preventive dental appointment.

Autoimmune thyroid disease has a detrimental effect on salivary glands and cause reduction in salivary secretion. ^12^^,^^16^ Basal metabolic rate is impacted by thyroid malfunction, and the secretary unit of the salivary duct is subsequently impacted. ^5^ Salivary secretion is reduced in AITD hypothyroidism. This is believed to be connected to the hypothyroidism-related decreased metabolism. Although the cause is unclear, AITD hyperthyroidism can reduce saliva production when it is untreated or only partly managed. ^17^

Salivary mucins have an important role in maintaining rheological properties of saliva. AITD impairs secretary unit of salivary gland and cause decreased quality of salivary mucins which in turn affects viscosity, pH and buffering capacity. ^18^

Farsi’s criteria have been used to evaluate subjective dryness among patients with thyroid disorders. A higher proportion of AITD subjects reported subjective dryness than in the control group, suggesting the impact of AITD on salivary flow. Unstimulated and stimulated saliva were significantly reduced in the AITD group which is similar to two previous studies, ^5^^,^^14^ suggesting consistent association between salivary flow and AITD. A higher proportion of study subjects in the AITD group experienced increased salivary viscosity and reduced salivary pH and buffering capacity, which was also similar to a previous study. ^5^ Hence, salivary parameters are significantly affected with patients with AITD.

A person with systemic hypothyroidism may have slower-healing cells and tissues, making them more vulnerable to illness. As a result, they are more likely to develop mouth and tooth conditions like dental caries, frequent mandible spasms, and bleeding lips. People who have autoimmune hyperthyroidism are more susceptible to dental caries, teeth sensitivity and pain in the jaw. ^13^ Autoimmune thyroid disease increases the risk of dental caries which has been attributed to the reduced salivary flow caused by the condition. ^19^ Dental caries experience was significantly higher in AITD group.

Indicating the efficacy of using Farsi’s criteria to assess oral dryness, a moderately significant negative association was discovered between “unstimulated salivary flow,” “stimulated salivary flow rate,” and “subjective oral dryness.” This indicates a connection between salivary flow and caries, and a marginally significant negative association was discovered between “unstimulated salivary flow,” “stimulated salivary flow rate,” and “dental caries.” Therefore, perceived dryness may be a sign of cavities development and salivary flow rate.

Dental caries was used as the result variable in a study using linear regression. In contrast to demographic factors, TSH, salivary viscosity, pH, and buffering capacity, which were not linked with dental caries, the current research discovered a highly significant correlation between salivary flow and dental caries.

The current research has some strengths and limitations. We believe that this research is the first of its kind to link salivary parameters and tooth caries in AITD. Standard kits and criteria were used to evaluate salivary parameters reflecting high internal validity.

Although it can be seen that there are risk factors, a cross-sectional research design does not allow for the evaluation of causation between study variables. Undiagnosed systemic disease could be present and could have affected the salivary metrics.

Prospective studies are recommended to understand changes in salivary parameters and the impact of drugs over a period of time among hypo and hyper AITD patients. There must be proper maintenance of oral hygiene and diet modifications.

Periodic dental management should be conducted by well trained dentists. Even if there are no comorbid conditions, dental therapy adjustments may be required for individuals who are receiving medical supervision and follow-up for an autoimmune thyroid disease.

Thyroid dysfunction can affect every system in the body, including the mouth. An overabundance or underabundance of these hormones has a negative impact on the oral cavity. The endocrinologist must be aware with the oral symptoms of thyroid dysfunctions before treating a patient with a thyroid disease. Prior to receiving dental care from a dentist, patients who have thyroid dysfunction and those who are on medication for it need to properly manage their risks. Thus, in order to preserve the patient’s thyroid and oral health, contact between the dentist and endocrinologist must be two-way.

## Conclusion

The current study may indicate an underlying association between thyroid and salivary gland dysfunction. Dental caries is a multi-factorial condition where diet and oral hygiene habits play key roles. Hence, future studies should take these factors into consideration to determine whether thyroid disorder alone can cause salivary gland dysfunction resulting in oral dryness and high dental caries experience.

## Data Availability

figshare: Association between dental caries experience and salivary profile among autoimmune thyroid disease subjects - a cross-sectional comparative study.xlsx, https://doi.org/10.6084/m9.figshare.23300891.v1. ^20^ This project contains the following underlying data:
- Term paper data entry.xlsx (underlying raw data file). figshare: Blank copy (proforma).docx, https://doi.org/10.6084/m9.figshare.23585322.v1. ^21^ This project contains the blank questionnaire and data collection form. figshare: Data key.xlsx, https://doi.org/10.6084/m9.figshare.23585319.v1. ^22^ This project contains the data key for the underlying data file. Data are available under the terms of the Creative Commons Zero “No rights reserved” data waiver (CC0 1.0 Public domain dedication).
