# Supplementary material for: Association between dental caries experience and salivary profile among autoimmune thyroid disease subjects - a cross-sectional comparative study
Source: F1000Res. 2023 Jul 14;12:833. [Version 1] doi: 10.12688/f1000research.135684.1 (PMC10721961; doi:10.12688/f1000research.135684.1)
Supplement: Peer review history

# Reviewer report: Reviewer response for version 1

**DOI:** 10.5256/f1000research.148815.r223547

**Published:** 2023-12-15

**Authors:** Amit Vasant Mahuli

**Affiliations:** 1 Department of Public Health Dentistry, Rajendra Institute of Medical Sciences, Ranchi, Jharkhand, India

Abstract: The abstract is well written and gives the gist of the article; however, the conclusion can be more specific with the results obtained.

 Introduction: It explains AITD and its relation to salivary secretions. It establishes the role of salivary secretions changes and their effects on oral health, thus establish the need for study 

 Methodology: It explicitly mentions sample size and sampling, inclusion and exclusion criteria, cases and controls, data collection, and a statistical plan.

 Results: It explains the differences between the AITD group and the control group and further explains the hyperthyroid and hypothyroid conditions.  Linear regression analysis with dental caries as a dependent variable shows a significant relationship between unstimulated and stimulated saliva. The author might have also considered the duration of diagnosis and duration of anti-thyroid drug usage, as they will influence the salivary secretion and process of dental caries.

 Discussion: The discussion is very well articulated and mentions study limitations and recommendations.

 Conclusion: The conclusion is more general in nature. The authors can be specific with the study results obtained, even though some results might not be statistically significant but indicate a difference.

Is the work clearly and accurately presented and does it cite the current literature?

Yes

If applicable, is the statistical analysis and its interpretation appropriate?

Yes

Are all the source data underlying the results available to ensure full reproducibility?

Yes

Is the study design appropriate and is the work technically sound?

Yes

Are the conclusions drawn adequately supported by the results?

Partly

Are sufficient details of methods and analysis provided to allow replication by others?

Yes

Reviewer Expertise:

Dental Public Health, Research, public health, Oral Cancer, Periodontal Disease

I confirm that I have read this submission and believe that I have an appropriate level of expertise to confirm that it is of an acceptable scientific standard.

# Reviewer report: Reviewer response for version 1

**DOI:** 10.5256/f1000research.148815.r223549

**Published:** 2023-12-14

**Authors:** Smitha B Kulkarni

**Affiliations:** 1 Dayananda Sagar College of Dental Science, Bengaluru, Karnataka, India

Title: Title is apt. Age group of the study subjects could have been mentioned.

 The introduction gives a detailed outline of the need for the study and the research questions along with objectives are clearly stated.

 Methodology: Inclusion and exclusion criteria are clearly stated. Sample size determination is clear.

 Methodology is explained in detail and gives a very good understanding of the study. Calibration and validation of the tools are explicitly mentioned Most of the confounding factors are eliminated.

 Statistical analysis is explained clearly.

 Results are well structured with good detailed explanation with tables related to all the parameters.

 Discussion is elaborate and conclusions are supported by the results.

Is the work clearly and accurately presented and does it cite the current literature?

Yes

If applicable, is the statistical analysis and its interpretation appropriate?

Yes

Are all the source data underlying the results available to ensure full reproducibility?

Yes

Is the study design appropriate and is the work technically sound?

Yes

Are the conclusions drawn adequately supported by the results?

Yes

Are sufficient details of methods and analysis provided to allow replication by others?

Yes

Reviewer Expertise:

Xerostomia related oral health problems, different mouthwashes for the treatment of gingivitis and prevention of dental caries

I confirm that I have read this submission and believe that I have an appropriate level of expertise to confirm that it is of an acceptable scientific standard.

# Reviewer report: Reviewer response for version 1

**DOI:** 10.5256/f1000research.148815.r191038

**Published:** 2023-08-21

**Authors:** Ridhima Gaunkar

**Affiliations:** 1 Department of Public Health Dentistry, Goa Dental College and Hospital, Bambolim, Goa, India

The introduction provides a sound outline of the research context stressing on the immune dysregulation in AITD and its effect on salivary characteristics and dental caries. The research question and objectives are clearly stated and open the way for subsequent sections of the article.

 The Methodology section is exhaustive and logically organized, providing a transparent understanding of the design of the study, methods of data acquisition and statistical analysis. The attention to calibration and validation of tools, ethical issues, explicit description of sample size estimation clearly enhances internal validity of the study.

 The results section is logically structured and provides detailed data analysis of variables related to demographics, dental caries, salivary parameters, thyroid function, correlations, and linear regressions.

 The discussion section adequately summarizes the study findings, provides relevant pointers from previous studies, and graciously acknowledges limitations. Its strength lies in suggesting prospective studies in future and a note on how dentists and endocrinologists can collaborate to address oral health problems in thyroid dysfunction patients.

 However, in the text description regarding anti-thyroid drugs (table 2), it would be better if the results are presented as 'less than one year' and 'one year and greater' instead of "The majority of the AITD subjects (81%) were under anti-thyroid drugs for the past 1 year." for better clarity and understanding.

 In table 7, the p value for DT (AITD group vs Control group should also be made bold) as it is statistically significant.

Is the work clearly and accurately presented and does it cite the current literature?

Yes

If applicable, is the statistical analysis and its interpretation appropriate?

Yes

Are all the source data underlying the results available to ensure full reproducibility?

Yes

Is the study design appropriate and is the work technically sound?

Yes

Are the conclusions drawn adequately supported by the results?

Yes

Are sufficient details of methods and analysis provided to allow replication by others?

Yes

Reviewer Expertise:

Epidemiology, Oral health promotion and disease prevention, Tobacco control, Research methodology and Statistics.

I confirm that I have read this submission and believe that I have an appropriate level of expertise to confirm that it is of an acceptable scientific standard.
